# Metallic Pin Causing Appendicitis: A Case From Syria

**DOI:** 10.7759/cureus.97483

**Published:** 2025-11-22

**Authors:** Yahya A Mutair, Roua Abdin, Mohamed Alabrash, Mohamed Issa, Hiba Rahhal

**Affiliations:** 1 Department of Surgery, Idlib University, Idlib, SYR; 2 Faculty of Medicine, University of Aleppo, Aleppo, SYR

**Keywords:** acute abdomen, appendicitis, metallic foreign body, radiology, urinary tract infection

## Abstract

Acute appendicitis is the most common surgical emergency, typically resulting from luminal obstruction caused by inspissated stool, lymphoid hyperplasia, vegetable seeds, parasites, or, rarely, foreign bodies. We report the case of a 10-year-old girl presenting with acute abdominal pain, nausea, and loss of appetite. Ultrasound and laboratory findings were diagnostic for acute appendicitis. Her previous urinary tract infection initially obscured the clinical picture, delaying suspicion of an alternative cause. The discovery of a metallic pin within the inflamed appendix highlights the importance of thorough evaluation when symptoms are atypical or recurrent. While CT scans are highly accurate for identifying ingested foreign objects, ultrasound remains a valuable radiation-free modality in pediatric patients. This case reinforces the need for prompt assessment of unusual abdominal symptoms and for considering foreign body ingestion in the differential diagnosis. It also underscores the importance of parental education to prevent accidental ingestion of sharp objects in children.

## Introduction

Acute appendicitis is one of the most common surgical conditions presenting to the emergency department. Symptoms may include abdominal pain, nausea, vomiting, and loss of appetite [1], and this condition affects all age groups. Pathophysiological mechanisms include retention of fecal debris, infection, tumors, and rarely foreign bodies [2]. When acute appendicitis is triggered by an ingested object lodged in the appendiceal lumen, it is referred to as foreign body appendicitis, a condition with only a few reported cases worldwide. Various metallic objects, such as screws, bird shots, and needles, have been documented, with bowel perforation occurring in approximately 1% of patients, particularly when sharp objects are involved [1].

We present the case of a 10-year-old girl with typical symptoms of appendicitis but a history of lower urinary tract infection, initially leading to diagnostic uncertainty. Ultrasound revealed a foreign body within the appendix, and abdominal surgery confirmed and removed a metallic object. This case report is presented in accordance with the Surgical CAse REport (SCARE) criteria, a standardized framework used to ensure clarity and quality in surgical case reporting [3].

## Case presentation

**Figure 1:**
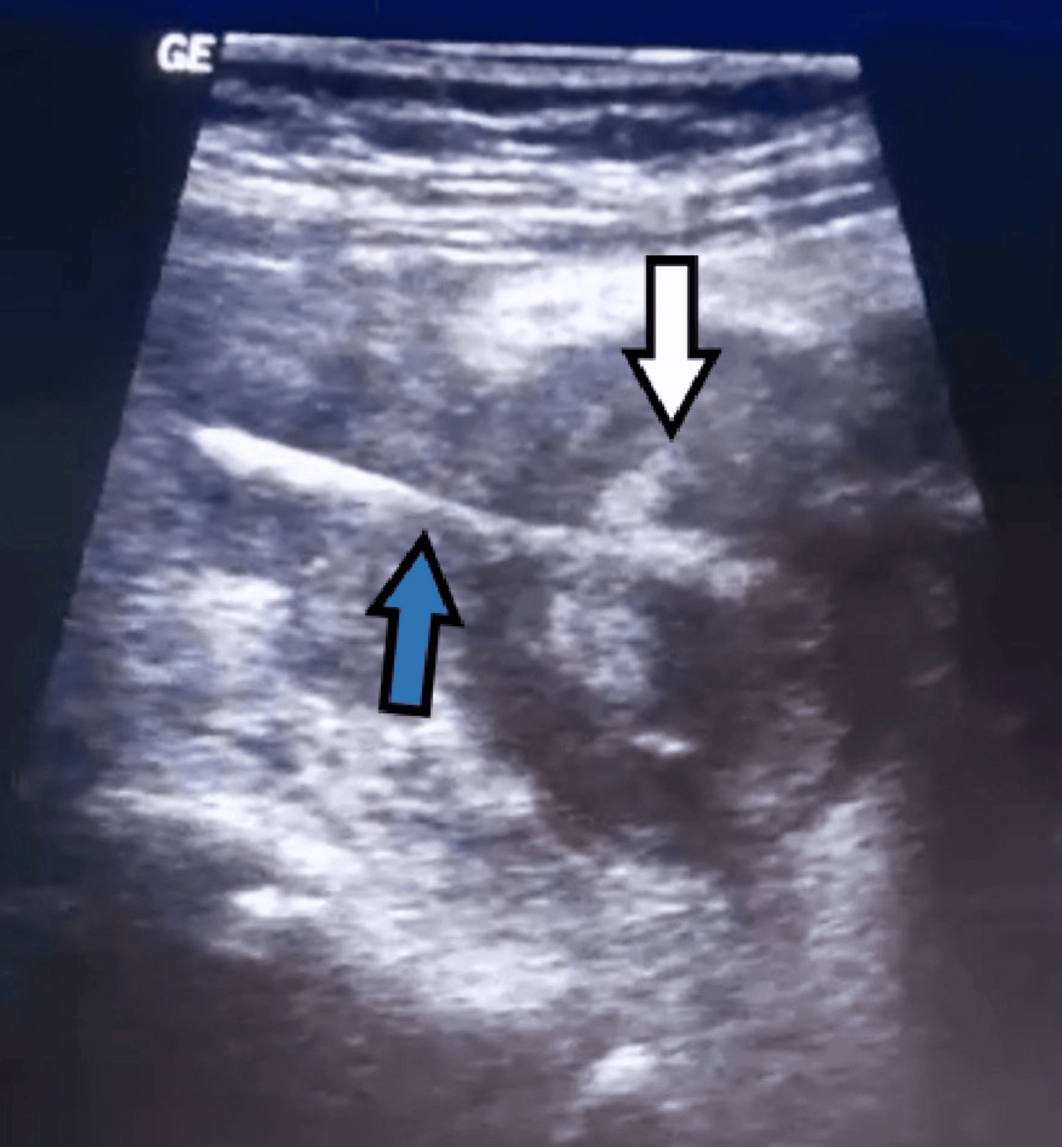
Ultrasound image showing a pin-shaped foreign object (blue arrow) located in the appendix (white arrow)

A 10-year-old girl came to a tertiary care hospital on 2024/11/11 to the emergency department with intermittent pain in the right lower quadrant that had lasted for several months, which became persistent and unresponsive to analgesics shortly before admission. Initially, the physicians suspected a urinary tract infection as the cause of her symptoms. However, on the night of her admission to the hospital, she experienced severe abdominal pain accompanied by nausea, a loss of appetite, and a low-grade fever. The patient's medical history included recurrent urinary tract infections for which she had been receiving appropriate treatment. Upon examination, tenderness was noted in the right lower quadrant with rebound tenderness, and clinical findings were suggestive of appendicitis. The girl had not received adequate attention from her parents, who were unaware that she had swallowed a pin. Laboratory studies revealed elevated white blood cell counts (Table 1). An ultrasound performed by the radiologist identified a foreign mass in the ileocecal junction with an unusual pin-shaped foreign body, with no free fluid observed (Figure 1).

**Table 1:** Laboratory blood test shows an increase in inflammatory cells, and the urinalysis shows pyuria and bacteriuria (+) Positive; (+++) Strongly Positive; HPF: High-Power Field

Test	Result	Normal Range
Blood		
White Blood Cells, cells/µL	14,000 (14 × 10^9^/L)	4,000-10,000 (4-10 × 10^9^/L)
Neutrophils, %	85	43-76
Lymphocytes, %	12	17-48
Urine		
Blood	(+)	Negative
Bacteria	(+++)	Negative
Epithelial Cells, cells/HPF	6-7	0-5

**Figure 2:**
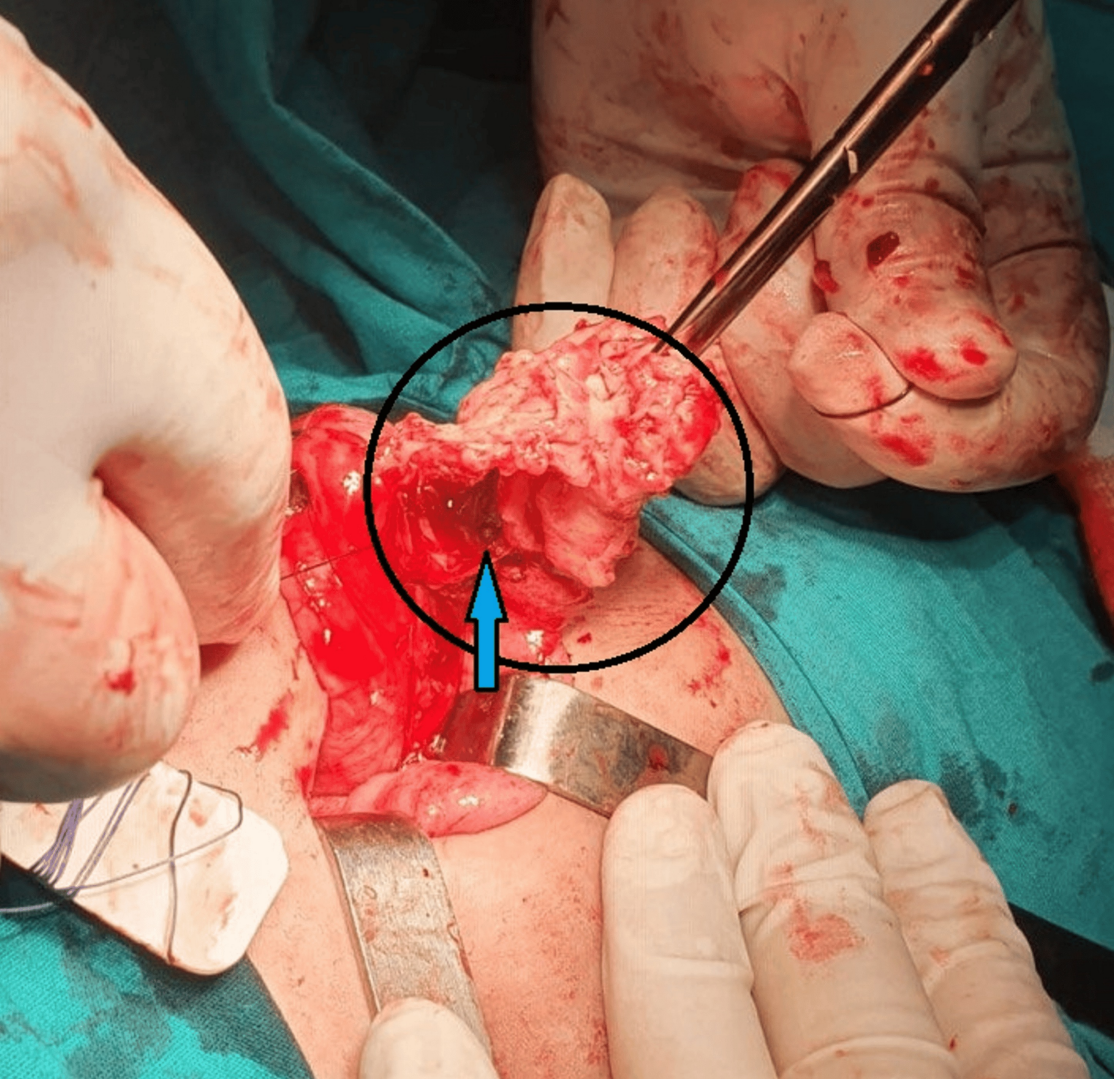
Intraoperative view highlighting the appendix The circular marking indicates the inflamed appendix, and the blue arrow points to the metallic pin embedded within the appendix.

**Figure 3:**
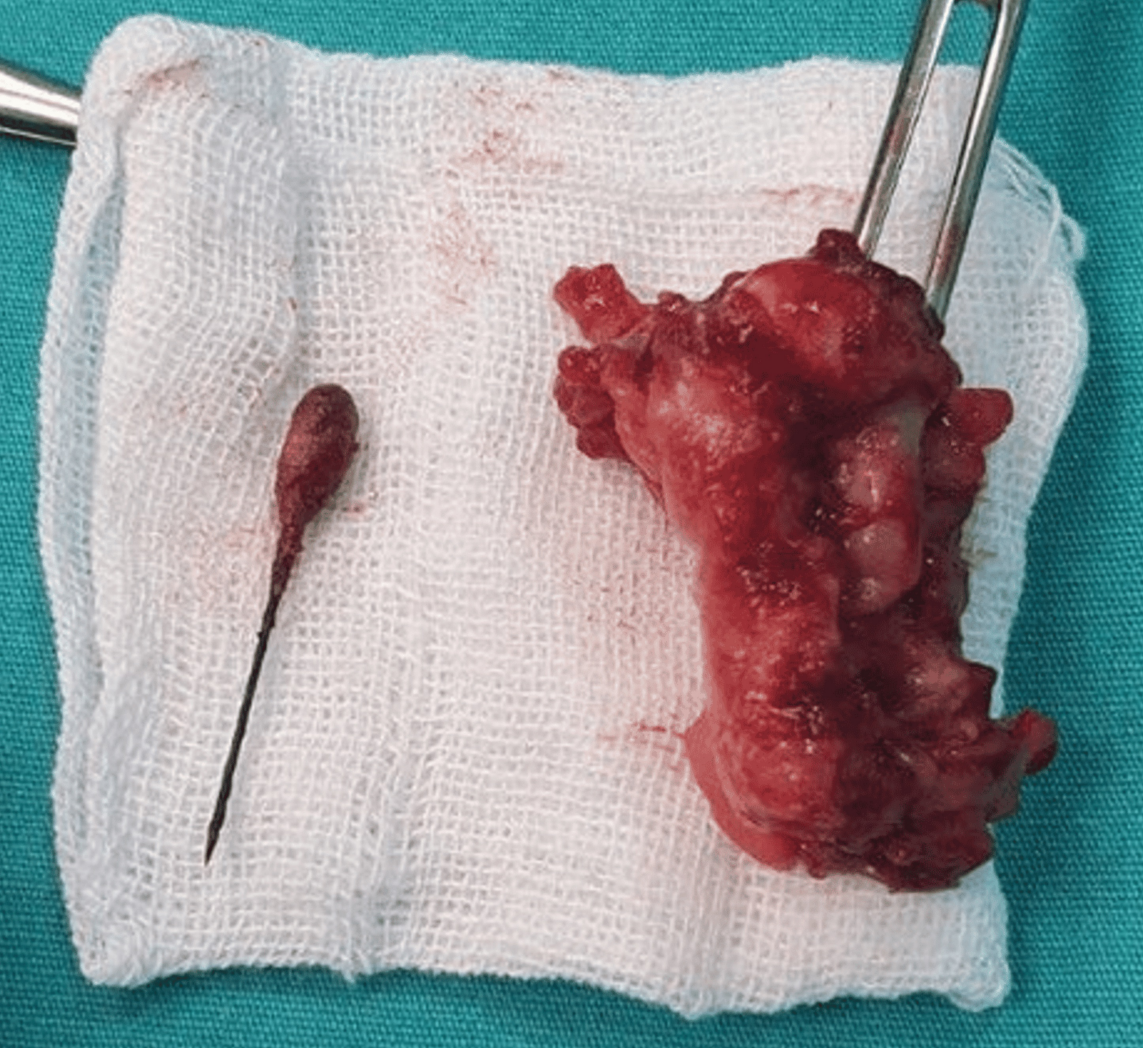
Postoperative image showing the removed appendix mass (phlegmonous mass) with the metallic pin clearly visible, confirming the source of the inflammation

Although CT is generally considered superior and can help rule out perforation, in the context of a low-resource setting, the diagnosis was confirmed with ultrasonography due to clear visualization. Then, surgical intervention was performed via a McBurney incision, revealing a mass in the terminal ileum, referred to as an appendix mass (phlegmonous mass). The incision was subsequently expanded to perform a Rutherford-Morrison approach, allowing for the removal of the mass (Figure 2), which included the base of the appendix and the pin (Figure 3).

Postoperatively, the patient experienced vomiting for two days but subsequently improved, and after three days of follow-up, the patient fully recovered.

## Discussion

Acute appendicitis is among the most frequent surgical issues encountered in the emergency department, typically presenting with fever, nausea, and abdominal pain, which begins peri-umbilical and then moves to the right lower quadrant [1]. In our case, a 10-year-old girl came to the hospital with ongoing pain in her right lower abdomen for several months. In fact, if no typical symptoms or physical irregularities are observed, making a diagnosis remains challenging [4]. In children, diagnosing the condition can take longer than in adults due to the presence of nonspecific symptoms [4]. Therefore, the patient’s condition was initially thought to be caused by a urinary tract infection. Upon her admission, she experienced intense abdominal pain, nausea, and a decreased appetite. The underlying causes of appendicitis include the accumulation of fecal matter, infections, tumors, and, less commonly, foreign bodies [1]. Although foreign bodies in the appendix are rare, they have been documented, with an estimated prevalence of 0.0005% [2]. It has been noted that long, sharp objects significantly increase the risk of perforation, while smaller objects can also lead to appendicitis due to obstruction [4]. Before the 20th century, appendicitis caused by foreign bodies was frequently observed. The earliest documented appendectomies occurred in 1735 and 1759, involving patients who had metal sewing pins that had perforated their appendices [5]. This is similar to what happened in our case; it was later discovered that she had accidentally swallowed a pin without her parents’ knowledge, which ultimately lodged in the appendix and caused obstruction and inflammation. Diagnosing foreign-body-related appendicitis is challenging, particularly in the absence of a clear history of ingestion. In such scenarios, abdominal X-rays are often of limited diagnostic value as they may not reliably identify foreign bodies and can lead to diagnostic confusion [5]. In this case, plain X-rays were not conducted, consistent with the recommendation to avoid their routine use in suspected appendicitis cases. Instead, an abdominal ultrasound was utilized, and it successfully identified a pin-shaped foreign object in the ileocecal junction, confirming the diagnosis. Ultrasound and CT scans are now considered the most effective imaging modalities for diagnosing appendicitis, with diagnostic laparoscopy being an alternative in certain cases [4]. Laboratory findings of an elevated white blood cell count also supported the diagnosis. Notably, the patient’s history of recurrent UTIs may have delayed the recognition of appendicitis, as the irritative pyuria caused by the inflamed appendix can mimic a true urinary tract infection. The treatment of acute appendicitis primarily involves the surgical removal of the vermiform appendix, which can be performed either via open surgery or laparoscopic techniques [1]. In this case, she underwent surgery using a McBurney incision, which was extended to a Rutherford-Morrison approach to extract the mass along with the base of the appendix and the pin. The patient developed a postoperative ileus, manifesting as vomiting for two days, which resolved with conservative management as evidenced by the return of bowel sounds and flatus.

## Conclusions

We presented a rare case of appendicitis caused by a metallic foreign body with a past medical history of urinary tract infection, which made the diagnosis more complicated. Children may swallow different objects when they are not under observation of their parents. An appendectomy was performed, and the entire mass was eradicated. This rare cause of acute appendicitis highlights the importance of considering foreign bodies as part of the etiology.
